# Probiotics in the Treatment of Chronic Rhinoconjunctivitis and Chronic Rhinosinusitis

**DOI:** 10.1155/2014/983635

**Published:** 2014-04-28

**Authors:** Matthias F. Kramer, Matthew D. Heath

**Affiliations:** Allergy Therapeutics plc., Dominion Way, Worthing, West Sussex BN14 8SA, UK

## Abstract

Chronic rhinitis and rhinosinusitis (CRS) are relevant health conditions affecting significant percentages of the western population. They are frequently coexisting and aggravating diseases. Both are chronic, noninfectious, and inflammatory conditions sharing to a certain extent important pathophysiologic similarities. Beneficial effects of probiotics are long known to mankind. Research is beginning to unravel the true nature of the human microbiome and its interaction with the immune system. The growing prevalence of atopic diseases in the developed world led to the proposition of the “hygiene hypothesis.” Dysbiosis is linked to atopic diseases; probiotic supplementation is able to alter the microbiome and certain probiotic strains have immunomodulatory effects in favour of a suppression of Th-2 and stimulation of a Th1 profile. This review focuses on randomized, double-blind, placebo-controlled trials investigating clinical parameters in the treatment of chronic rhinitis and CRS. An emerging number of publications demonstrate beneficial effects using probiotics in clinical double-blind placebo-controlled (dbpc) trials in allergic rhinitis (AR). Using probiotics as complementary treatment options in AR seems to be a promising concept although the evidence is of a preliminary nature to date and more convincing trials are needed. There are no current data to support the use of probiotics in non-AR or CRS.

## 1. Chronic Rhinoconjunctivitis and Chronic Rhinosinusitis

ARIA guideline defines rhinitis as a chronic inflammatory disease of the nose resulting in nasal symptoms including nasal obstruction, sneezing, and anterior or posterior rhinorrhea (occurring during two or more consecutive days for more than one hour) [1].

Allergic rhinitis (AR) is the most common form of noninfectious, chronic rhinitis affecting more than 25% percent of the European population [1, 2]. It is characterized as an eosinophilic, IgE-mediated, Th-2 dominated immune disorder. “Local allergic rhinitis” describes a condition of local allergen-specific IgE production in the nose. Prevalence data are estimated to lay between 47% and 62.5% of patients with perennial and seasonal symptoms. Interestingly, this condition is described to precede a “classic” AR [3].

Prevalence data about nonallergic forms of chronic, noninfectious rhinitis are rare. They are estimated to be almost as high as AR [1]. Non-AR includes a long list of potential causes. However, the idiopathic form remains the most frequent [4]. Although non-AR is per exclusion not a type-I allergy it resembles often the same cellular key players such as mast cells and eosinophils [5].

EPOS guidelines define chronic rhinosinusitis (CRS) by the presence of at least two of the following symptoms for at least 12 weeks per year: nasal blockage, nasal discharge, facial pain or pressure, or reduction of smell with at least one of the symptoms being nasal blockage or nasal discharge.

Chronic rhinosinusitis can occur with or without nasal polyps (CRSwNP or CRSsNP) [6].

The pathophysiology of CRS is only partially understood. It is characterized as a chronic inflammation resembling components of Th-2 (eosinophils, mast cells) and Th-1 immune responses [6–8].

Chronic rhinitis and CRS are frequently coexisting and aggravating conditions. Both are chronic, noninfectious, and inflammatory conditions sharing important pathophysiologic similarities such as a Th-2 type immune pattern.

## 2. Probiotics

Beneficial effects of probiotics are long known to and practiced by mankind. The Russian immunologist Metschnikow (Nobel  prize  laureate 1908) published the results of extensive studies about probiotics in his book “The Prolongation of Life” in 1907. Present day, the human microbiome concept and probiotics are experiencing an impressive renaissance.

A probiotic widely consists of a food product or supplement containing sufficient numbers of viable microorganisms aimed at altering the microflora of the host and, in turn, conferring beneficial health effects. The World health Organisation describes* Probiotics* as “live microorganisms which, when administered in adequate amounts, confer a health benefit on the host.” Probiotic microorganisms are typically consumed in fermented foods (i.e., cheeses, yoghurts) and most commonly used* genera* include* Lactobacilli* and/or* Bifidobacterium*. They are typically anaerobic organisms and in the intestines they ferment ingested food products to produce lactic acid. Their inherent biological features enable them to predominate and prevail over potential pathogenic microorganisms in the human digestive tract.

*Prebiotics* are nondigestible food ingredients selectively stimulating the favorable growth and/or activity of probiotics. Prebiotics are usually oligosaccharides such as fructooligosaccharides (FOSs), inulin, or galactooligosaccharides (GOSs).

*Synbiotics* are the combination of probiotics and prebiotics [9].

## 3. The Microbiome and Dysbiosis

Our understanding of the human microbiome and its interaction with the human immune system is increasing rapidly. A PubMed search of the term probiotics presents thousands of citations during the past 10 years, compared to less than 100 for the previous 25 years. The intestine—the largest lymphoid organ in the body—and particularly the large intestine is heavily colonized and commensal bacteria outnumber human cells by a factor of 10 to 1. The intestinal microbiome plays a key role in the maintenance of mucosal health [10] and research continues to present an intensive crosstalk at play between this interface. In addition to this, the skin (the largest human organ) consists of a densely populated and diverse habitat of microbiota. Research is only just beginning to unravel the unprecedented influence this equally complex and dynamic ecosystem plays in the onset and progression of a number of chronic inflammatory diseases [11].

A disrupted microbiome (= dysbiosis) has been associated with a lengthening list of health conditions such as obesity and malnutrition, diabetes, numerous diseases of the intestines, autism, and chronic inflammatory conditions such as atopy or rhinitis [12, 13].

Subsequent to the sterile uterine environment, colonization begins at birth. By one (three) year(s) of age the microbiome has a stable adult-like signature [12, 14, 15]. Thus, postnatal microbiome development is thought to play a pivotal role in infant health. The type of delivery (vaginal or cesarean section) undoubtedly contributes to the ratio of colonized* genera *as a result of different exposures during delivery, their effects of which may persist for a period of time after birth [16]. For example, infants born from a cesarean section have been linked to higher risk categories for some immune-mediated diseases [17, 18]. Life events such as travelling, antibiotics, short/long term dietary changes, and illnesses will alter the composition of the microbiome [19, 20].

There is little doubt that the influence of probiotic bacteria has the ability to exert indirect or direct immunomodulatory effects, however their detailed mechanisms remain to be determined. Other mechanisms of action continue to be observed which include modulation of cellular metabolism and epithelial barrier functions. Interestingly, many specific effects and efficacy have been shown to be species or strain specific [21–26].

The interactions of probiotics with the host immune system are only partially understood and include, for instance, the following.

- *Humoral immunity:* stimulation of Th 1, suppression of Th 2, stimulation of Tregs, transforming growth factor *β* and an increase in local IgA production which influences mucosal defences [21, 22, 27].
- *Innate immunity/adjuvant effects:* toll-like receptor signalling (TLR-2), nucleotide-binding oligomerization domain receptors (NODs)- or lectins signaling, and interaction with dendritic cells (modulation of DC maturation and their cytokine patterns) [28, 29]. Additional interactions of the microbiome and the human body are executed via the “gut-brain-axis” [12, 30]. Furthermore, probiotics can serve as mucosal delivery vehicles, exhibit a “colonization resistance” by their commercial properties, and enhance the epithelial gut barrier [28]. However, mechanistic studies are mostly based on* in vitro* cell models and make it difficult to translate or accurately portray native* in vivo* mechanisms.

Supplementation of pre- or probiotics is unlikely to resolve conditions in predisposed individuals predominated by complex genetic factors and/or sever dysregulation of their immune system. However, the association of certain mild-severe diseases linked to microbiome dysbiosis may offer realistic prophylactic or therapeutic treatment options. The beneficial effects of probiotic consumption in a variety of inflammatory diseases (e.g., irritable bowel disease, chronic respiratory diseases) have been reported [31, 32]. Due to aforementioned characteristics, it is obvious that probiotics can be studied for beneficial effects in the prevention and treatment of chronic rhinitis and CRS.

## 4. Dysbiosis and AR

The growing prevalence of atopic disease in the developed world led to the proposition of the “hygiene hypothesis” by Strachan in 1989 [33]. In the progression of that early hypothesis the crucial role of microbial environmental stimuli for atopy was emphasized advancing it into the “microbial hypothesis” [34]. Considering the collective genomes of microbes that live inside and on us, in addition to our own, one has engineered the term: the human supraorganism to describe our true form [14]. Human evolution has brought the industrialization of the modern world and, with it, advances in technology which have transformed people's lives over the past century. More importantly, such environmental changes play a fundamental role in altering our biosphere, thus our health and onset and progression of diseases. The rise of atopic eczema in industrialized countries has now reached epidemic levels within the last five decades [35].

Dysbiosis could conclusively be linked to atopy—in animal studies [36] and man [37–39].

## 5. Probiotics and Prevention of Atopy

Probiotics that are tailored and marketed towards treating individuals suffering from a range of atopic diseases are starting to emerge and grow on the market.

Using probiotics for prevention of atopic diseases was initiated by Scandinavian trials published in high-impact journals demonstrating significant effects in the prevention of atopic dermatitis [40–42]. Here,* Lactobacillus (L.) rhamnosus* appears to be a primary candidate strain in the incidence of atopic dermatitis. However, overall data is conflicting and evidence is limited [43]. Human studies can be very difficult to compare since they can vary considerably in their design (i.e., screening, duration, clinical end-point definitions, etc.). Recent reviews see moderate effects in the prevention of atopic dermatitis (in subgroups) but not in AR, asthma, or allergic sensitizations [44, 45]. Interestingly, a recent investigation examined associations between consumption of probiotic milk products in pregnancy and infancy with questionnaire-reported atopic eczema, rhinoconjunctivitis, and asthma in 40,614 children. In this population-based cohort the consumption of probiotic milk products was related to a reduced incidence of atopic eczema and rhinoconjunctivitis, but no association was seen for incidence of asthma by 36 months of age [46]. In addition to this, a study performed by Kuitunen and colleagues [17] provided a strong hypothesis in that babies, delivered via caesarean section, who received synbiotics had fewer IgE-associated diseases (24.3%) compared to a placebo group (40.5%) at the age of 5 years. Much needed data is necessary to confirm or refute this hypothesis, since this study also concluded contradictory results in which the incidence of atopic disease in 925 neonatal infants, who each received synbiotics, was comparable to a placebo group after 2 and 5 years.

The complex crosstalk and array of effects by which prebiotics and probiotics elicit are not fully understood and this may explain, in part, why results of human studies, which use synbiotics to induce immune-health benefits, have been contradictory [47]. However, study designs are under increasing scrutiny and the need to better define validated biomarkers, valuable enough to substantiate a health claim, has yet been achieved.

## 6. Treatment of Chronic Rhinitis and CRS by Probiotics

### 6.1. Allergic Rhinitis

As explained above, dysbiosis is linked to atopic diseases, probiotic supplementation is able to alter the microbiome, and certain probiotic strains have immunomodulatory effects in favour of a suppression of Th-2 immune response and stimulation of Th-1 and Tregs. Hence, there is an objective rationale for studying probiotics in the treatment of AR. Over the last years an emerging number of randomized, dbpc trials focusing on clinical data in humans were published for the treatment of AR.

#### 6.1.1. Seasonal AR

Wassenberg et al. studied* L. paracasei* in a dbpc cross-over trial (*n* = 31) versus placebo in grass pollen allergic patients in 2011 by means of nasal provocation (NPT) over 4 weeks of treatment. Nasal congestion in NPT was significantly improved by active treatment [48].

Ouwehand et al. analyzed the combination of* L. acidophilus *and* Bifidobacterium (B.) lactis* in 47 children suffering from birch pollen AR in a dbpc trial versus placebo over 4 months. The combination of the selected probiotics was shown to prevent the pollen-induced infiltration of eosinophils into the nasal mucosa and indicated a trend for reduced nasal symptoms [49].

*B. longum* significantly improved eye symptoms in 40 patients with allergic rhinoconjunctivitis due to Japanese cedar in a dbpc setting versus placebo over 14 weeks. Nasal symptoms improved as well, although not statistically significant [50, 51].

*B. lactis* was studied in 20 patients suffering from grass AR in a dbpc trial against placebo over 8 weeks during the grass pollen season. Total nasal symptom score improved significantly. IL-5, IL-13, and TNF-alpha were significantly decreased, likewise was the CD63 expression on activated basophils [52].

Lastly, Perrin et al. studied* L. paracasei *versus a combination of* L. acidophilus* and* B. lactis* in 31 grass pollen allergic patients in a dbpc cross-over design over 4 weeks.* L. paracasei* significantly reduced nasal pruritus while not affecting nasal congestion in that setting [32].

#### 6.1.2. Perennial AR

Wang et al. analyzed* L. paracasei* in 80 patients suffering from house dust mite (HDM)-allergic rhinitis in a dbpc trial versus placebo over 30 days. Scores for the overall quality of life significantly decreased in the* L. paracasei *group as compared against placebo, in both frequency and level of bother [53].

*L. acidophilus* was analyzed in 49 HDM-allergic patients against placebo in a dbpc trial for 8 weeks. Administration of* L. acidophilus* resulted in a statistically significant improvement of nasal symptom-medication scores [54].

12-week treatment of* L. salivarius* reduced rhinitis symptoms and drug usage in 240 children suffering from HDM-AR against placebo in a dbpc trial [55].

Lin et al. conducted a 12-week dbpc trial using an interesting design: 60 children with perennial AR were randomized into two groups with 28 participants receiving levocetirizine plus placebo and 32 participants receiving regular levocetirizine plus* L. paracasei* for the first 8 weeks, with a shift to levocetirizine as rescue treatment during the following 4 weeks. The* L. paracasei* group had significantly lower Pediatric Rhinoconjunctivitis Quality of Life Questionnaires (PRQLQ) scores even after discontinuing regular levocetirizine from week 9 to week 12. There was more improvement in individual parameters in the PRQLQ including: sneezing, itchy nose, and swollen puffy eyes, in the active group. The authors summarized that dietary supplementation with* L. paracasei* provided no additional benefit when used with regular levocetirizine in treating AR in the initial 8 weeks of our study, but there was a continuing decrease in PRQLQ, as well as a significant improvement in individual symptoms of sneezing, itchy nose, and swollen eyes, after discontinuing regular levocetirizine treatment [56].

The above listed publications demonstrate beneficial effects using probiotics in clinical dbpc trials in AR. Many questions remain open: duration of treatment, strain selection, optimal dosage of strains, potential additional positive effects using prebiotics, and so forth. Due to the limited number of published trials and factors such as “publication bias” these data are of preliminary nature to date. However, effects have been shown to be reproducible and more clinical trials will be conducted. Using pre-, pro-, or synbiotics as complementary treatment options in AR seems to be a promising concept.

Interestingly, there is an increasing body of evidence in animal models revealing future options: probiotics can provide beneficial effects for immunotherapy [57] or recombinant probiotics, producing IL-10 or allergens such as Bet v1 or HDM-allergens, could have the potential for novel treatment options for AR [58–61]. The real power of probiotics may lie in the use of genetically modified lactic acid bacteria. For example, evidence from these studies indicates that deletions to certain cell surface components can ultimately downregulate inflammatory responses [62]. However, such alterations to cell surface components of lactic acid bacteria inevitably call into question their GRAS (“generally regarded as safe”) status [47].

### 6.2. Nonallergic Rhinitis

To the author's best knowledge there exists no trial focusing specifically on non-AR.

### 6.3. CRS

Mukerji et al. analysed the oral use of* L. rhamnosus* on sinonasal quality-of-life scores in CRS. 77 patients were studied in a dbpc trial against placebo over a 4-week treatment, revealing no significant results [63].

However,* Staphylococcus (Staph.) aureus* is a key pathogenic component of the CRS microbiome and is associated with increased disease severity and poor postoperative outcomes. Cleland et al. investigated the probiotic properties of* Staph. epidermidis* against* Staph. aureus* in a mouse model of sinusitis. They confirmed the probiotic potential of* Staph. epidermidis* in that model [64].

Biofilms form on moist biotic and abiotic surfaces, making them common for infections of the ears, nose, and throat and especially in CRS. Eradicating ENT biofilms is difficult. Probiotics such as* L. casei* were shown to have beneficial effects in ENT biofilms [65].

Furthermore, upper respiratory tract infections (URTI) are often preceding CRS. The use of probiotics in URTI was summarized in a Cochrane review in 2011, stating that “probiotics were better than placebo in reducing the number of participants experiencing episodes of acute URTIs, the rate ratio of episodes of acute URTI and reducing antibiotic use” [66].

Recently the same could be demonstrated for healthy physically-active adults. West et al. found a significantly reduced risk of URTI using* B. lactis* in a dbpc trial including 464 subjects over 150 days of treatment [67].

Hence, published evidence does not support the use of probiotics in CRS to date. However, more data are required to finally address the question whether probiotics are beneficial in CRS.

## 7. Conclusion

The paradigm of the human microbiome and the relationship of dysbiosis and distinct diseases is a fascinating concept attracting increasing attention. However, there is a requirement for more consistent data from human studies and a better understanding in their mode of action through* in vitro/in vivo* models to answer many remaining open questions. It is widely demonstrated that baseline variation exists amongst the population; non-responders are frequently reported and this may be dictated by specific and ill-defined phenotypic factors. However, through understanding the role and importance of host-dependent (e.g., genetic background, diet and lifestyle, innate microflora compositions etc.) factors, may provide the opportunity to design more personalised treatment programmes designed to confer improved clinical efficacy for specific individuals or sub-populations [47]. In addition, generating sufficient scientific evidence to support a health claim may well be achieved through a better understanding of immune phenotypes of individuals and how this dictates the immunomodulatory effects elicited through the supplementation of synbiotics.

Preliminary data exist providing beneficial results in using probiotics in the treatment of allergic rhinitis and probiotics could emerge as a novel, complementary treatment option for AR. However, there are no current data to support the use of probiotics in non-AR or CRS.
